# Employee–Organization Relationships and Team Performance: Role of Team Collective Efficacy

**DOI:** 10.3389/fpsyg.2020.00206

**Published:** 2020-03-06

**Authors:** Juexing Li, Liangding Jia, Yahua Cai, Ho Kwong Kwan, Shuyang You

**Affiliations:** ^1^Department of Business Administration, School of Management, Nanjing University, Nanjing, China; ^2^Department of Human Resource Management, School of Business, Shanghai University of Finance and Economics, Shanghai, China; ^3^Department of Organizational Behavior and Human Resource Management, China Europe International Business School (CEIBS), Shanghai, China; ^4^Department of Business Administration, School of Business Administration, Dongbei University of Finance and Economics, Dalian, China

**Keywords:** employee–organization relationship, collective efficacy, team cohesion, team performance, human resource management

## Abstract

Besides the previous social relationship perspective of employee–organization relationship (EOR) research, this study takes the social cognitive perspective to explore the role of team collective efficacy in mediating the relationship between EORs and team performance. This study further contends that team cohesion moderates the positive relationship between collective efficacy and team performance, thereby moderating the indirect relationship between EORs and team performance through collective efficacy. Data analyses of 231 teams in Study 1 and 63 teams in Study 2 support the hypotheses. Therefore, this study provides theoretical contributions to the EOR literature by introducing a new perspective at the team level and to the social cognitive literature by discussing a boundary condition of the effect of collective efficacy on team performance.

## Introduction

In recent decades, the competitive environment has dramatically changed employment relationships in organizations (Jia et al., 2014; Cai et al., 2016). Lifetime employment systems have been replaced by numerous employment relationship forms from which organizations can select their preferences to increase employment flexibility (Tsui and Wang, 2002; Andrijasevic and Sacchetto, 2017). Understanding diverse employment relationships has practical importance in the contemporary business world, thereby attracting the attention of human resources scholars over the past decades (Eldor and Vigodagadot, 2017).

**FIGURE 1:**
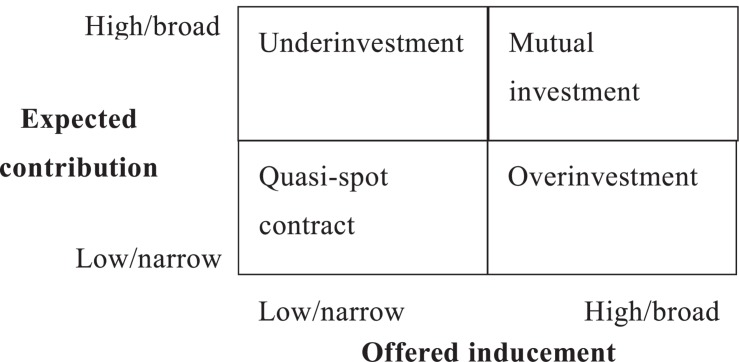
Typology of employee–organization relationship approaches.

From employers’ perspective, an employment relationship should include inducements that employers provide employees [i.e., offered inducements (OIs)] and employers’ expectation of contributions from employees in return [i.e., expected contributions (ECs)] (Tsui et al., 1997). By configuring these two dimensions, Tsui et al. coined four types of employee–organization relationships (EORs), including two balanced EORs, namely, (1) quasi-spot contract (low OI and low EC) and (2) mutual investment (high OI and high EC). Typical examples are piece workers in contract manufacturers and product managers in Internet startups, respectively. The other two types are unbalanced EORs, namely, (3) underinvestment (low OI and high EC) and (4) overinvestment (high OI and low EC) (Tsui et al., 1995; Tsui et al., 1997). Figure 1 shows the typology of EOR approaches.

Prior studies have explored how EORs shape social exchange relationships between employees and organizations (Shaw et al., 2009; Cai et al., 2016), job-embeddedness relationships between middle managers and organizations (Hom et al., 2009), and information-exchange relationships among team members (Jia et al., 2014) to influence attitudes, performance, and creativity. These studies have shown a consensus conclusion that among the four types of EORs, mutual investment has the highest effect in predicting individual attitudes (e.g., employee and middle manager commitment, trust, and turnover), performance, organizational citizenship behavior (OCB), and firm performance (Wang et al., 2003; Jia et al., 2014; Cai et al., 2016).

Two important research gaps remain despite intriguing progress. First, previous EOR studies have focused on the effect of EORs in shaping social relationships among different social agents, thereby influencing their attitudes, behaviors, and performance. However, such studies ignore the characteristics of agents themselves. “To be an agent is to intentionally make things happen” (Bandura, 2001). Social agents are actors who take actions to participate in social activities, including individuals, teams, organizations, governments, and so on. Aside from relationships among agents, the characteristics of agents also play important roles in influencing their performance. Social cognition, as an important characteristic, reflects agents’ perception, interpretation, and response to the environment as well as their cognition about themselves, which in turn influences their behavior (Bandura, 1997, 2001). Several scholars have appealed to future studies to explore the cognitive process associated with EORs and team outcomes (Jia et al., 2014). Therefore, to fill the gap in the literature, we shift attention from social relationships among agents to the characteristics of the agents themselves, thereby exploring the mechanism of EORs on performance from the social cognitive perspective. Efficacy beliefs are the most persuasive, pivotal, and important constructs in social cognitive theory (Bandura, 1997, 2009). Thus, we choose efficacy beliefs as a proxy for the social cognitive perspective to investigate its mediating effect between EORs and performance.

Second, EORs have been largely conceptualized as an organization-level human resource management (HRM) practice, while less attention has been paid to their variability at the team level. Given that work teams have increasingly become the building blocks of organization management, investigating the effect of team-level EORs on team performance is necessary (Mathieu et al., 2008; Rego et al., 2017; Rodríguez-Sánchez et al., 2017). Generally, organizations will not treat teams the same way but will complement specific HRM practices according to the specific functions and importance of the teams. Nonetheless, different supervisors may understand, transfer, and implement the same EOR policy differently (Den Hartog et al., 2013). For example, organizations will separately provide R&D, production, and marketing teams different compensation packages and require different contributions according to the teams’ idiosyncratic functions. In theory, considering team-level HRM practices and treating EORs as team-level constructs are also important and reasonable. Human resource architecture literature suggests that different teams are subject to different employment relationships in a firm, and research to capture these differences is needed (Lepak and Snell, 1999). Strategic HRM researchers have also pointed out that this literature area has not given adequate attention to the variability within organizations, thereby calling for team-level HRM studies (Chang et al., 2014). Actually, the original conceptualization of EORs is anchored at the job level wherein firms adopt multiple approaches of EORs (Tsui et al., 1997). Jia et al. (2014) also proposed and tested the rationality and possibility of studying EORs at the team level. To fill this gap and respond to the call for research, we discuss and measure EORs at the team level to explore how team-level EORs influence team performance through team collective efficacy. Team collective efficacy represents team-level efficacy beliefs and refers to the shared beliefs of team members on their joint ability to organize and implement actions to attain a certain level of achievement (Bandura, 1997). Social cognitive theory and previous research have indicated that collective efficacy is the most pivotal emergent state through which team input (i.e., EOR in this study) affects team performance (Bandura, 1997; Chen et al., 2002; Mcleod and Orta-Ramirez, 2018).

Furthermore, given the identified heterogeneous effects between collective efficacy and team performance across studies (Stajkovic et al., 2009), we postulate that providing clarity on what contexts would affect this relationship is theoretically important. Since collective efficacy affects only team members with group-oriented values, we introduce team cohesion as a contingent factor that moderates the relationship between collective efficacy and team performance (Schaubroeck et al., 2000; Khong et al., 2017). Team cohesion refers to the degree to which team members are attracted to one another in pursuing a common objective (Harrison et al., 1998). Cohesive teams with high collective efficacy will be more dedicated to team tasks and persevere longer in the face of adversity. Therefore, team cohesion strengthens the relationship between collective efficacy and team performance and thus the indirect relationship between EORs and team performance through collective efficacy.

**FIGURE 2:**
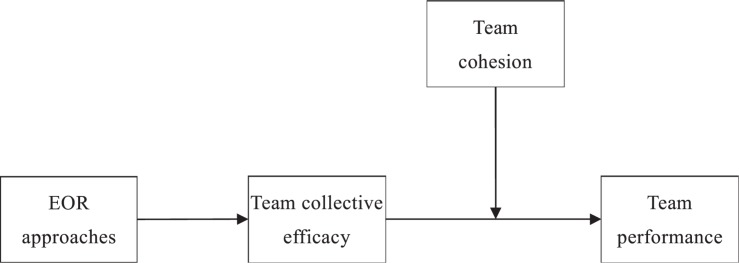
Social cognitive perspective of team-level employee–organization relationship (EOR), team collective efficacy, team cohesion, and team performance.

Taken together, we theorize the relationship between EORs and team performance through team collective efficacy and examine team cohesion as a moderator. We postulate that OI and EC will jointly enhance the emergence of team collective efficacy by providing the four information sources of efficacy beliefs, that is, enactive mastery experience, vicarious experience, social persuasion, and physiological and emotional states (Goddard et al., 2004; Bandura, 2009). Thus, a mutual investment EOR approach, with high OI and high EC, will generate the highest team collective efficacy and lead to the highest team performance. Furthermore, team cohesion will strengthen the relationship between collective efficacy and team performance and likewise strengthen their indirect link. A moderated mediation model is shown in Figure 2. We conduct two studies to test our hypotheses. Specifically, Study 1 collects data from 231 knowledge-intensive teams in 55 Chinese high-tech organizations, while Study 2 collects data from 77 supervisors and 305 team members.

We make three contributions to the literature. First, in addition to the previous social relationship perspective of EOR research, we study the effect of EORs on performance from the social cognitive perspective by exploring the mediating role of team collective efficacy. Second, we go beyond macro- and micro-level studies that treat EORs as organizational HRM practices and focus on meso-level team EORs and team performance. Thus, new insights into team-level employment relationships will be a valuable extension to the HRM literature. Third, we contribute a culture-free contingent factor to extend social cognitive theory by introducing team cohesion as a moderator between team collective efficacy and performance.

## Theoretical Background and Hypotheses

### EORs

From employers’ perspective, previous HR researchers have focused on how organizations treat their employees, that is, what inducements are provided to employees, such as a high-commitment work system (Arthur, 1994; Whitener, 2001). However, organizations also want contributions from employees. Thus, integrating inducement and contribution dimensions into a coherent theoretical framework is necessary to capture the full picture of employment relationships.

Tsui et al. (1997) are the first to combine the inducements provided by an organization to employees with the contributions that the organization expects from employees to build EOR theory. Drawing on the inducement–contribution framework (Barnard, 1938; March and Simon, 1958) and social exchange theories (Blau, 1964), the authors come up with an EOR typology. Specifically, OIs are employer-provided material and developmental rewards. Narrow, low OIs are short-term and purely economic rewards. However, broad, high OIs include generous material rewards, long-term career development investments, and attention to employee well-being. Meanwhile, organizations expect their members to reciprocate by contributing their work efforts. On the one hand, employers who expect low contributions will explicitly stipulate well-specified and close-ended job requirements in contracts and job descriptions. On the other hand, employers who expect high contributions will ask employees to “adopt permeable and expandable work roles” (Tsui et al., 1997) and encourage a wide range of extra-role behaviors and open-ended obligations (Cai et al., 2016).

Tsui et al. (1997) identified two balanced and two unbalanced EOR approaches, as shown in Figure 1. One balanced approach is the quasi-spot contract EOR, in which employers provide specific and narrow material rewards and demand employees to meet basic task requirements. A typical example of this approach is the pure economic exchange relationship between piece workers and contract manufacturers. The second balanced approach is the mutual investment EOR, in which employers provide generous rewards and invest in long-term career development while expecting employees to engage in various pro-organization or pro-team activities and contribute to the entire organization beyond individual functions (Wang et al., 2003). Presently, numerous Internet startups use the mutual investment approach to build relationships with their venture partners and high-quality professionals. OI and EC are asymmetric in the two unbalanced approaches. In the underinvestment approach, employers offer relatively low and narrow rewards but expect broad contributions. Pare (1989) described that many companies use this approach to manage their middle managers owing to the heavy pressure during the 1980s (Wang et al., 2003). By contrast, in the overinvestment approach, OIs are extensive, whereas ECs are limited. Certain jobs in the government belong to this approach.

Researchers have discussed the effects of EORs on different relationships among agents. Moreover, they used social exchange theory to explore reciprocal relationships between organizations and employees and found that the mutual investment approach yields the best employee performance and attitudes (Shaw et al., 2009; Song et al., 2009). Researchers have also discussed embedded relationships between organizations and middle managers (Hom et al., 2009) as well as communication relationships among team members (Jia et al., 2014).

However, few EOR studies have explained how EOR approaches, specifically, mutual investment, shape agents themselves. In this study, we shift the research focus from relationships among social agents to the agents themselves, namely, work teams. Drawing on social cognitive theory, we discuss the role of team collective efficacy in linking EORs to team performance.

### EORs and Collective Efficacy

We choose collective efficacy as the socio-cognitive proxy between EORs and team performance for three reasons. First, human agency is the foundation of social cognitive theory, and efficacy beliefs are its most central, pivotal, and persuasive mechanisms (Bandura, 1997, 2000, 2009). Second, collective efficacy is an emergent property that captures the joint efficacy beliefs of teams as a whole; thus, it is suitable to represent team-level social cognitive mechanisms. Third, collective efficacy is empirically more salient in predicting team performance than self-efficacy (Stajkovic et al., 2009; Khong et al., 2017). Meta-analysis shows that team-level collective efficacy, rather than individual self-efficacy, is more strongly related to team performance (Gully et al., 2002).

According to social cognitive theory, efficacy beliefs are state-like, malleable, and subject to idiosyncratic contextual influences of four principal sources of information, including mastery experience, vicarious experience, social persuasion, and physical and emotional states (Luthans et al., 2007; Bandura, 2009). This framework is used commonly in the literature to explain the development of collective efficacy (Cybulski et al., 2005). Following the literature, we also use the framework to argue that team collective efficacy is higher in teams under the mutual investment EOR approach than in teams under other EOR approaches, because the combination of OI and EC will play a positive role in providing the four sources of information.

First, enactive mastery experiences are acquired through successes and failures and provide authentic and direct evidence of capabilities (Bandura, 1997). Mutual investment EORs can improve team collective efficacy by providing opportunities to navigate difficult tasks to enable members to acquire adequate enactive mastery experiences. When teams are asked and motived to handle various conditions, confront difficult tasks, and meet challenges, they will have increased experiences and knowledge of rules as well as strategies for effective daily work and thus high beliefs in their collective efficacy (Chen et al., 2002). However, without the help of generous inducements, team members may feel pressured to engage in work, which in turn will decrease their mastery experience (Bakker et al., 2014). Researchers find that team members will have the highest level of work engagement when both job demands and resources are high (Bakker et al., 2007, 2014). Therefore, when teams are under the mutual investment EOR approach, with high levels of OI and EC, members will engage in work and thus have increased opportunities to master experiences.

Second, vicarious experiences are acquired by observing and learning from others’ experiences to gain competence (Bandura, 1997). When teams are trained in knowledge and skills for career development, they have opportunities to learn from experts, consultants, experienced supervisors, and coworkers (Wang et al., 2003; Bandura, 2009). Consequently, they would be able to manage resources and complete tasks under challenging conditions (Bruton et al., 2016). Meanwhile, long-term relationships allow teams to observe other teams to acquire task-relevant vicarious knowledge that will increase their collective efficacy (Bandura, 1997).

Third, social persuasion occurs when the positive beliefs or feedback of others generates self-confidence. Mutual investment can act as social persuasion for communicating expectations and rewards to members (Goddard et al., 2004). By expecting high-level contributions from a team, an organization expresses its confidence in the team’s capacity and potential by encouraging its members to achieve ambitious goals and discouraging them from giving up when encountering difficulties (Goddard et al., 2004). As a result, their shared beliefs about their competence are strengthened, thereby triggering high collective efficacy (Bandura, 1997; Chen et al., 2002; Bandura, 2009; Wu et al., 2010). Meanwhile, high inducements act as indirect persuasive signals, as they communicate organizational confidence that teams have potential and deserve generous rewards as well as long-term investment. In addition, according to Goddard et al. (2004), career development opportunities and feedback on achievement, which are typical practices in mutual investment, can act as social persuasion to inspire collective efficacy. Team members will develop collective confidence in their competence when they perceive this persuasive signal. From their sample of 109 elementary schools, Ross and Gray (2006) found that principals can increase teachers’ collective efficacy by offering visionary and inspirational messages.

Fourth, when team members experience highly activated somatic physiological and emotional states, such as being healthy and relaxed and feeling positive, they are likely to develop collective efficacy (Goddard et al., 2004). High rewards generate positive physiological and emotional states, which in turn influence team collective efficacy directly through perceived capabilities and indirectly through selection, interpretation, and information recall (Bandura, 1997). When team members share positive physiological and emotional states, they positively evaluate their situations and thus form high collective efficacy.

In summary, the mutual investment approach will elicit the greatest team collective efficacy. By contrast, the quasi-spot contract approach will generate the lowest team collective efficacy, as it denies teams necessary mastery experiences, vicarious experiences, persuasion, and material and psychological rewards. Prior studies on collective efficacy have provided some indirect evidence for our argument. For example, empowerment, which is a component of OI, is positively related to collective efficacy (Jung and Sosik, 2002). Studies on the influence of transformational leadership on collective efficacy have also suggested that members will develop collective efficacy when encouraged to venture beyond standard expectations (Ross and Gray, 2006; Kurt et al., 2012). Formally, we propose the following.

Hypothesis 1: The quasi-spot contract EOR approach yields the lowest team collective efficacy, the mutual investment EOR approach yields the highest collective efficacy, and the overinvestment and underinvestment EOR approaches yield in-between levels of collective efficacy.

### Mediating Effect of Collective Efficacy

“The higher the sense of collective efficacy, the better the team performance” (Bandura, 1997). Teams with high collective efficacy will share strong beliefs that they can jointly accomplish tasks and enjoy future success; hence, they tend to set ambitious team goals (Chen et al., 2002; Wu et al., 2010). Moreover, they are confident that they can allocate, coordinate, and integrate resources in specific situations (Bruton et al., 2016). In addition, they are motivated to put effort into teamwork while managing and integrating resources and skills efficiently. When teams encounter obstacles, collective efficacy allows members to become resilient and help one another recover from hindrance work stressors (Khong et al., 2017). A meta-analysis has shown the positive relationship between collective efficacy and team performance (Gully et al., 2002).

Social cognitive theory demonstrates that efficacy beliefs mediate the effect of external stimuli on outcomes. For example, students attending schools with high socioeconomic status and academic pressure will have high collective efficacy, which can lead to high mathematics achievements (Hoy et al., 2002). Management scholars have also indicated that collective efficacy mediates the relationships between transformational leadership and group effectiveness (Jung and Sosik, 2002) and between high-performance HR practices and team creativity (Ma et al., 2017). Combining the above logic, we propose that team collective efficacy will mediate the effects of EORs on team performance.

Hypothesis 2: The relationship between EOR approaches and team performance is mediated by team collective efficacy.

### Moderating Effect of Team Cohesion

In cohesive teams, members develop strong psychological bonds with one another as well as with the team (Severt and Estrada, 2015). In addition, they are committed to their team and shared goals (Rodríguez-Sánchez et al., 2017). Team cohesion will augment the relationship between collective efficacy and team performance by triggering the cognitive convergence of team collective efficacy beliefs (Park et al., 2017). When team cohesion is high, members are committed to team goals rather than individual goals (Mathieu et al., 2015). Thus, their attention will focus on collective aspects, such as collective efficacy, thereby motivating them to activate and strengthen the collective cognition process (Schaubroeck et al., 2000). Hence, they can easily transfer collective beliefs into positive interactions, collective actions, and persistence to generate high team performance (Park et al., 2017; Rodríguez-Sánchez et al., 2017).

By contrast, when team cohesion is low, team members lack motivation to pursue team goals jointly. Despite believing that they can function together effectively, team members may put individual interests above team goals (Rodríguez-Sánchez et al., 2017). Thus, they will not be able to reach agreements and look forward to different objectives (Chang et al., 2014). In the absence of psychological bonds and collective identification, few team members are willing to help others or stick together when faced with adverse situations. Thus, we argue that low team cohesion will weaken the relationship between collective efficacy and team performance because team performance is not the simple summary of individual performance but requires team members to complete tasks together. Hence, we propose the following.

Hypothesis 3: Team cohesion moderates the positive relationship between team collective efficacy and team performance such that the positive relationship will become stronger when team cohesion is higher.

In addition, we form a joint moderated mediation framework of the relationships between EOR approaches, team collective efficacy, team cohesion, and team performance.

Hypothesis 4: Team cohesion moderates the indirect relationship between EORs and team performance through team collective efficacy: higher team cohesion will strengthen the indirect relationship, and the mutual investment approach will have the highest effect on team performance via collective efficacy under higher team cohesion.

## Materials and Methods

### Study 1

#### Research Design and Procedure

This study is part of a large project involving a two-wave on-site survey of high-technology companies in an eastern province of the People’s Republic of China (PRC). First, we randomly selected 102 organizations from 2,043 high-technology organizations listed on the official website of the Ministry of Science and Technology of the PRC. Next, we sent letters to the top managers of the selected organizations to explain our research goals and obtain permission. We also visited HR directors to learn about organizational structures to choose the most suitable teams and employees to participate in the survey as well as to discuss survey schedules. Then, we randomly selected 5 to 10 teams per company from a list of work teams provided by the HR directors. The functions of the selected teams included R&D, product design, technical support, manufacturing, quality testing, and customer service. All the participants were full-time employees.

The HR directors helped administer the on-site surveys. They gathered the participants in company meeting rooms, and we gave each participant a business card, a gift, and a cover letter explaining the questionnaires and our commitment to confidentiality. The HR directors agreed to deliver the business cards, gifts, questionnaires, cover letters, and self-addressed and stamped return envelopes to the few absent participants. The *t*-test of our core variables showed no significant differences between the 90% of surveys completed on-site and those returned by mail.

We implemented two-wave on-site surveys after a pilot survey in seven companies to improve our research procedures. In the first wave, we measured OI, EC, team collective efficacy, team cohesion, and an important control variable, namely, perceived supervisor support. We measured team performance 2–4 months later in the second wave.

#### Sample

A total of 65 of the 102 randomly selected organizations participated in the first-wave survey (64% participation rate). The respondents included 307 team supervisors and 2,317 employees. Each organization averaged 4.72 teams (ranging from 1 to 7) and 35.65 employees (ranging from 2 to 93). A total of 55 out of the 65 organizations participated in the second wave (85% rate). The respondents included 239 team supervisors and an average of 4.35 teams from each organization. We tested response bias in the two waves and found that teams participating at Time 2 did not differ significantly from teams in the first wave in terms of team size, supervisor age, gender, education, or company tenure (see Supplementary Appendix 1).

In the 307 teams, the average within-team response rate was 96% (from 46.7 to 100%). After matching usable cases of key variables, we tested the hypotheses using a sample of 1,800 employees from 231 teams of 55 organizations. In the final sample, on average, the supervisors were between the ages of 36 to 40 years, 77.1% were men, mean education level was junior college, and average tenure in their current position was 6.6 years. Each team averaged 7.79 employees.

#### Measures

##### EORs

We adapted items measuring OI and EC from Hom et al. (2009) to Wang et al. (2003). The items were answered on a Likert-type scale ranging from 1 (*seldom provided*) to 7 (*provided a lot*) for OI and from 1 (*seldom emphasized*) to 7 (*emphasized very much*) for EC, 0 = *not existing* for both. To ensure that the items were appropriate for capturing team-level employment relationships, we interviewed HR directors and conducted an exploratory factor analysis and confirmatory factor analysis (CFA).

We asked supervisors to rate team-level EORs, as they have knowledge on the content and extent of the inducements that their organizations offer to teams as well as the contributions that their organizations expect from teams by interpreting, transferring, and implementing firm HRM practices. This procedure has also been used in recent studies to measure team- or department-level HRM practices (Van De Voorde and Beijer, 2015; Lin et al., 2019). First, we asked the supervisors to indicate the job titles and number of members in the teams and provide a brief job description before rating the OI and EC of the teams so that they can keep the entire team in mind. For OI, we asked, “To what extent does your firm provide the following inducements to the group of employees…” A total of 14 OI items followed the question, such as “emphasize employees’ career development,” “care about employees’ satisfaction at work,” and “provide competitive salaries.” Cronbach’s alpha was 0.91. For EC, we asked, “To what extent does your firm emphasize the following expected contributions from the group of employees…” and then followed the question with 13 items, such as “fulfill the job inside and out,” “team up with others in the job,” and “continuously improve work procedures and methods.” Cronbach’s alpha was 0.94. Scales of mian variables are shown in Supplementary Appendix 2.

Following the classic approach of Tsui et al. (1997), we used a median split to create an approximation of the four types of EORs according to the supervisors’ ratings on OI and EC. When the two dimensions scored below the median, we identified a team as having the quasi-spot contract approach. We identified a team as having the underinvestment approach when OI scores were below the median but EC scores were above or equal to the median. Furthermore, we identified a team as having the overinvestment approach when OI scores were above or equal to the median but EC scores were below the median. Finally, we identified a team as having the mutual investment approach when the two dimensions scored above or equal to the median.

Table 1 shows the details of the four EOR approaches. In the final sample, the 231 teams were divided into four categories, that is, 75 teams had quasi-spot contract EOR, 39 teams had underinvestment EOR, 29 teams had overinvestment EOR, and 88 teams had mutual investment EOR. ANOVA results indicated that OI and EC differed significantly across the categories (*F* = 131.093, *p* < 0.001 and *F* = 118.427, *p* < 0.001, respectively).

**TABLE 1:** Study 1: EOR categories.

	EOR: team level					ANOVA	
	Quasi-spot contract	Under investment	Over investment	Mutual investment	Total mean	*F*-value	*p*-value
Offered inducements	4.0254 (−0.8262)	4.2623 (−0.6074)	5.4322 (0.4731)	5.8679 (0.8756)	4.9439	131.093	0.000
Expected contributions	5.0971 (−0.9517)	6.5256 (0.7129)	5.4733 (−0.5134)	6.5385 (0.7279)	5.9346	118.427	0.000
N of team	75	39	29	88	231		

*EOR, employee–organization relationship. Numbers in parentheses are means of standardized scores.*

##### Team collective efficacy

Eight items of collective efficacy were adapted from Riggs and Knight (1994) and Salanova et al. (2003). Adaptation was necessary, as a few original items were reverse-scored, which may cause common method bias and could disappear after being changed into positively worked ones (Podsakoff et al., 2003). The team supervisors rated the groups’ collective efficacy as a whole. Although aggregation of the perception of every member is common in the literature, asking supervisors to rate the team-level constructs was feasible. Two reasons may allow them to provide unbiased or trained ratings (Salas et al., 2015). First, team supervisors have sufficient knowledge on entire teams through everyday observations and interactions with team members. Second, by circumventing the need to estimate the perception of every team member, external observation is an unobtrusive way to measure collective constructs. An example item is, “I feel confident about the collective capability of this group to perform tasks very well,” which was answered on a six-point Likert-type scale ranging from 1 (*strongly disagree*) to 6 (*strongly agree*). Cronbach’s alpha was 0.88.

##### Team cohesion

We used items from Harrison et al. (1998) to measure team cohesion: “Overall, the group of employees (1) is ready to defend each other when facing criticism by outsiders, (2) helps each other well on the job, (3) gets along well with each other, and (4) sticks together well.” We also asked each supervisor to rate team cohesion as an external observer using a six-point Likert-type scale ranging from 1 (*strongly disagree*) to 6 (*strongly agree*). This method was also used by Chang et al. (2014). The reliability of the four items was 0.57. We dropped the first item owing to its very low or negative correlations with the other three items (i.e., 0.006, -0.023, and -0.028). The final Cronbach’s alpha was 0.88.

##### Team performance

Five items of team performance were adopted from Barrick et al. (1998): (1) quantity of work, (2) quality of work, (3) work planning and allocation, (4) knowledge of tasks, and (5) overall performance, which were answered on a five-point Likert-type scale ranging from 1 (*below average*) to 5 (*above average*). Cronbach’s alpha was 0.80.

##### Control variables

We controlled for several demographic variables as potential antecedents of collective efficacy and team performance, including *team size*, members’ *average age*, *female percentage*, *average education*, and *average team tenure*. Team size was measured by the number of employees in a team. For age, 1 = less than 25 years, 2 = between 26 and 30 years, 3 = between 31 and 35 years, 4 = between 36 and 40 years, 5 = between 41 and 45 years, 6 = between 46 and 50 years, 7 = between 51 and 55 years, 8 = between 56 and 60 years, and 9 = above 60 years. For education, 1 = middle school or below, 2 = technical or high school, 3 = junior college, 4 = bachelor’s degree, 5 = master’s degree, and 6 = doctorate degree. In addition, we controlled for *perceived supervisor support* as a representative of social exchange theory. The seven-item scale was developed by Pearce et al. (1992).

#### Analyses

We performed CFA on our major variables by running five-factor (our hypothesized measurement model), four-factor, three-factor, two-factor, and one-factor CFA. Table 2 shows that the five-factor model fits the best (χ*^2^* = 1713.999, *df* = 850, χ*^2^/df* = 2.016, RMSEA = 0.068, NFI = 0.918, CFI = 0.957, IFI = 0.957).

**TABLE 2:** Study 1: Results of CFA.

Model	χ*^2^*	*df*	χ*^2^/df*	RMSEA	NNFI	CFI	IFI
5-factor: OI, EC, CE, TC, TP	1,713.999	850	2.016	0.068	0.918	0.957	0.957
4-factor: OI + EC, CE, TC, TP	2,387.120	854	2.795	0.119	0.886	0.923	0.923
3-factor: OI + EC, CE, TC + TP	2,736.784	857	3.193	0.129	0.869	0.906	0.906
2-factor: OI + EC + CE, TC + TP	3,334.539	859	3.882	0.151	0.840	0.876	0.876
1-factor: OI + EC + CE + TC + TP	3,631.615	860	4.223	0.162	0.826	0.861	0.862

*CFA, confirmatory factor analysis; OI, offered inducement; EC, expected contribution; CE, collective efficacy; TC, team cohesion; TP, team performance.*

We tested whether the data suffered from common method variance (CMV) using Harman’s single-factor test. The result showed that the first unrotated factor can explain only 30% of the total variance, which meant that the common method bias may not be problematic. In addition, Siemsen et al. (2010) demonstrated that CMV cannot create an artificial interaction effect but only weaken existing interactions. Therefore, the authors pointed out that CMV need not be considered when testing moderating effects. If we find a significant interaction effect between team cohesion and collective efficacy on team performance, then the result will provide strong evidence for its existence. We also used the WarpPLS software developed by Kock (2019) to perform a full collinearity test to check whether our model suffered from CMV. According to Kock (2017), if all VIFs in the full collinearity test are equal to or less than 3.3, then the model is free of common method bias. All full collinear VIFs in Study 1 were less than 2.2, thereby suggesting that our model did not suffer from CMV.

The 231 teams were nested in 55 organizations; thus, teams in the same organizations were interdependent, which violated the fundamental independent assumption underlying traditional ordinary least squares regression. We used STATA to conduct clustered regression, with a White correction that allows covariance among individuals within groups and corrects for heteroscedasticity across groups (Rogers, 1993). Standard errors were adjusted for 55 organization clusters in the clustered regression.

#### Results

Table 3 shows the correlations and descriptive statistics. The quasi-spot contract approach was negatively and significantly correlated with team collective efficacy and team performance. Moreover, the mutual investment approach was positively and significantly correlated with team collective efficacy and team performance. Meanwhile, team collective efficacy was positively and significantly correlated with team performance.

**TABLE 3:** Study 1: Correlations and descriptive statistics.

	Mean	SD	(1)	(2)	(3)	(4)	(5)	(6)	(7)	(8)	(9)	(10)	(11)	(12)
(1) Team size	7.79	4.37												
(2) Average age	2.82	1.09	–0.12											
(3) Female percentage	1.66	0.28	0.07	0.03										
(4) Average education	3.09	0.73	0.04	−0.46^∗∗^	0.10									
(5) Average team tenure	4.06	3.32	–0.11	0.65^∗∗^	–0.09	−0.33^∗∗^								
(6) Perceived supervisor support	4.59	0.54	–0.03	0.10	–0.06	–0.11	–0.02							
(7) Quasi-spot contract	0.32	0.47	–0.03	0.05	0.11	0.05	0.03	–0.06						
(8) Underinvestment	0.17	0.38	0.01	–0.08	−0.13^∗^	0.09	–0.01	−0.13^∗^	−0.31^∗∗^					
(9) Overinvestment	0.13	0.33	0.02	0.04	–0.04	–0.04	–0.05	0.11	−0.26^∗∗^	−0.17^∗∗^				
(10) Mutual investment	0.38	0.49	0.02	–0.01	0.03	–0.09	0.01	0.08	−0.54^∗∗^	−0.35^∗∗^	−0.30^∗∗^			
(11) Team collective efficacy (Time 1)	5.12	0.56	–0.04	0.00	–0.03	–0.04	0.08	0.01	−0.34^∗∗^	–0.05	–0.03	0.39^∗∗^		
(12) Team cohesion (Time 1)	5.27	0.69	–0.11	0.00	–0.12	–0.08	0.11	0.09	−0.29^∗∗^	0.02	–0.01	0.27^∗∗^	0.58^∗∗^	
(13) Team performance (Time 2)	3.92	0.56	0.04	–0.01	–0.01	0.03	0.02	0.09	−0.17^∗∗^	0.03	–0.09	0.20^∗∗^	0.33^∗∗^	0.18^∗∗^

*p < 0.05 and **p < 0.01; *N* = 231.

Table 4 shows the clustered regression analysis results. As shown in Step 2, team collective efficacy was significantly higher in teams with underinvestment, overinvestment, or mutual investment EOR approaches than in teams using the quasi-spot contract approach, and the coefficients increased (β = 0.20, *p* = 0.09; β = 0.25, *p* < 0.05; β = 0.56, *p* < 0.001, respectively). In addition, we ran another model with the mutual investment approach as the base category. The model showed that teams with quasi-spot contract, underinvestment, and overinvestment approaches had significantly lower collective efficacy than teams with a mutual investment approach, and the absolute values of the coefficients decreased (β = −0.56, *p* < 0.001; β = −0.36, *p* < 0.01; β = −0.31, *p* < 0.01, respectively). Therefore, the highest team collective efficacy occurred under the mutual investment EOR (high OI and EC), while the lowest occurred under the quasi-spot contract EOR (low OI and EC), thereby supporting Hypothesis 1. Although we did not distinguish between the effects of underinvestment (low OI and high EC) and overinvestment (high OI and low EC) EOR approaches, the results indicated that the overinvestment approach was better than the underinvestment approach in generating collective efficacy. The finding suggested that OI had a stronger effect on collective efficacy than EC.

**TABLE 4:** Study 1: Hierarchical regression analysis results.

	Team collective		Team performance			
	efficacy (Time 1)		(Time 2)			
	Step 1	Step 2	Step 3	Step 4	Step 5	Step 6
Constant	5.16^∗∗∗^	4.89^∗∗∗^	3.87^∗∗∗^	3.74^∗∗∗^	3.81^∗∗∗^	3.74^∗∗∗^
Team size	–0.00	–0.01	0.01	0.01	0.01	0.01
Average age	–0.06	–0.04	–0.02	–0.00	0.01	0.00
Female percentage	–0.01	–0.01	–0.01	0.00	0.00	0.02
Average education	–0.03	0.01	0.03	0.05	0.05	0.04
Average team tenure	0.02^†^	0.02^†^	0.01	0.01	0.00	0.00
Perceived supervisor support	0.02	–0.02	0.10	0.10	0.11	0.12^†^
**EOR (Time 1)**						
Underinvestment		0.20^†^		0.19	0.13	0.14
Overinvestment		0.25^∗^		–0.01	–0.08	–0.06
Mutual investment		0.56^∗∗∗^		0.28^∗∗^	0.11	0.11
Team collective efficacy (Time 1)					0.31^∗∗∗^	0.34^∗∗∗^
Team cohesion (Time 1)						0.00
Team collective efficacy * team cohesion						0.27^∗∗^
R^2^	0.02	0.19^∗∗∗^	0.01	0.07^†^	0.14^∗∗∗^	0.18^∗∗∗^
ΔR^2^		0.17^∗∗∗^		0.05^∗∗^	0.08^∗∗∗^	0.04^∗∗^
F	0.97	6.62^∗∗∗^	0.45	1.81^†^	5.11^∗∗∗^	5.28^∗∗∗^
ΔF		15.85^∗∗∗^		4.08^∗∗^	19.24^∗∗∗^	4.85^∗∗^

^†^*p* < 0.1, *p < 0.05, **p < 0.01, and ***p < 0.001; *N* = 231. Δ*F* = (Δ*R*^2^/Δ*k*)(*N*−*k*_2_−1)/($1-R_{2}^{2}$) *k* is the number of predictors, and *N* is the sample size (Jaccard et al., 1990).

Table 4 wformance (β = 0.31, *p* < 0.001). Furthermore, when team collective efficacy was added to the equation in Step 5, the coefficients of the mutual investment approach were no longer significant. Table 5 shows the results of the mediation test of team collective efficacy between EOR approaches and team performance using the Monte Carlo method. Compared with the quasi-spot contract approach, the underinvestment approach had a non-significant indirect effect on team performance through team collective efficacy (the 95% confidence interval included 0), and the overinvestment and mutual investment approaches had significant indirect effects on team performance through team collective efficacy (the 95% confidence intervals did not include 0). Thus, team collective efficacy mediated the relationship between EORs and team performance, supporting Hypothesis 2.

**TABLE 5:** Study 1: Mediation test using Monte Carlo Method–Hypothesis 2.

				95% confidence	
	a	b	a × b	interval	
Underinvestment	0.20	0.31	0.06	−0.01	0.14
Overinvestment	0.25	0.31	0.08	0.01	0.16
Mutual investment	0.56	0.31	0.17	0.10	0.26

*The mediator is team collective efficacy.*

**FIGURE 3:**
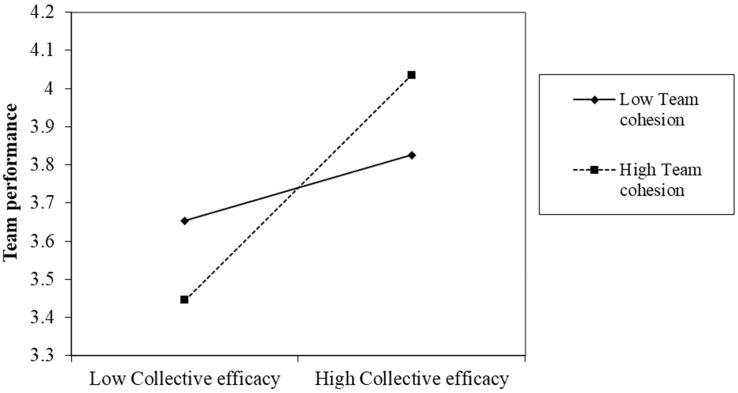
Moderating effect: team collective efficacy on team performance at low and high levels of team cohesion–Hypothesis 3.

In the last column of Table 4, team collective efficacy positively and significantly interacted with team cohesion to affect team performance (β = 0.27, *p* < 0.01), thereby explaining an additional 4% of the variance. The coefficient of team cohesion on team performance was small and non-significant (β = 0.00, *p* = 0.99), which may be weakened by the single-rater method. However, as discussed above, the interaction effect was also weakened, and thus, the significant interaction effect in the results became conservative and credible (Siemsen et al., 2010). The 95% confidence interval of the path analysis in Table 6 shows that team collective efficacy had a non-significant effect on team performance at low team cohesion levels but a positive and significant effect at high team cohesion levels. The two effects differed significantly across team cohesion levels. Figure 3 shows the relationships between team collective efficacy and team performance at low and high levels of team cohesion, supporting Hypothesis 3, which predicted that team cohesion would have a moderating effect.

**TABLE 6:** Study 1: Path analytic results–direct, indirect, and total effects of EOR Approaches on team performance (via team collective efficacy) at low and high levels of team cohesion (95% confidence interval)–Hypothesis 4.

					Direct effects		Indirect effects		Total effects	
	P_MX_		P_YM_		(P_YX_)		(P_YM_ × P_MX_)		(P_YX_ + P_YM_P_MX_)	
**a–Underinvestment**										
Simple paths for low team cohesion	–0.05	0.43	**−0.04**	**0.32**	–0.09	0.34	−0.01	0.12	−0.06	0.39
Simple paths for high team cohesion	–0.05	0.43	**0.32**	**0.77**	–0.09	0.34	−0.02	0.25	−0.01	0.49
**b–Overinvestment**										
Simple paths for low team cohesion	0.04	0.46	**−0.04**	**0.32**	–0.28	0.13	**−0.00**	**0.13**	**−0.24**	**0.18**
Simple paths for high team cohesion	0.04	0.46	**0.32**	**0.77**	–0.28	0.13	**0.03**	**0.29**	**−0.17**	**0.31**
**c–Mutual investment**										
Simple paths for low team cohesion	0.39	0.71	**−0.04**	**0.32**	–0.08	0.28	**−0.02**	**0.19**	**0.01**	**0.37**
Simple paths for high team cohesion	0.39	0.71	**0.32**	**0.77**	–0.08	0.28	**0.16**	**0.47**	**0.20**	**0.60**

**N* = 231; Confidence intervals in bold are significantly different across team cohesion levels. The base category is the quasi-spot contract approach. P_*MX*_, path from X (underinvestment in a, overinvestment in b, mutual investment in c) to M (team collective efficacy); P_*YM*_, path from M to Y (team performance); P_*YX*_, path from X to Y.*

Table 6 shows the results of the path analysis used to test the moderated mediation hypothesis (i.e., Hypothesis 4). When the quasi-spot contract approach was the base category, the underinvestment approach had non-significant direct, indirect, or total effects on team performance via team collective efficacy at low, high, or across team cohesion levels. However, the overinvestment approach had a significant indirect effect only under high team cohesion. The overinvestment approach had significantly different indirect and total effects on team performance through team collective efficacy across team cohesion levels. Meanwhile, the mutual investment approach had significant indirect effects at high team cohesion levels and significant total effects at both team cohesion levels. Finally, the mutual investment approach also had significantly different indirect and total effects on team performance through team collective efficacy across team cohesion levels. In summary, team cohesion moderated the indirect relationships between EOR approaches and team performance via team collective efficacy. Thus, Hypothesis 4 was supported.

#### Discussion

In Study 1, we test and support all the hypotheses using data from 231 teams. Our findings suggest that team-level EORs will facilitate team performance by developing team collective efficacy. Team cohesion will moderate this indirect link by influencing the relationship between team collective efficacy and team performance. Collective efficacy will have a positive effect on team performance only when team cohesion is high. Thus, the indirect effects of EOR approaches are significant only when team cohesion is high.

Nevertheless, though the dependent variable is measured at Time 2, CMV may still exist, as all of the main variables are rated by team supervisors. Therefore, we conduct a new study to test whether EOR approaches can influence team performance via collective efficacy when we collect data from multiple sources and multiple time periods. In other words, we will test whether our main findings from Study 1 are influenced by CMV.

### Study 2

#### Research Design and Procedure

The participants of Study 2 are MBA students and their subordinates from a top business school in China. One of the researchers first asked the MBA students to rate the OI and EC and their demographic information at Time 1. Next, they were asked to invite five subordinates from their teams to take part in the survey at Time 2. These team members rated their perception of team collective efficacy and demographic information. Finally, the MBA students rated team performance at Time 3. All the questionnaires were electronic and sent via the Internet.

#### Sample

After the three-wave survey, we obtained a sample of 77 supervisors and 305 team members. On average, four members comprised each team participating in the survey. For the team supervisors, 69.4% were male, and their highest education levels were junior college and above, with most having a master’s degree (72.2%). Their age ranged from 27 to 51 years, with a mean of 35.99 years. As for the team members, 58% were male, and their age ranged from 21 to 60 years, with a mean of 33.69 years. Most of the team members had a bachelor’s degree (62.6%), and average work tenure was 6.75 years. After matching, our final data for hypothesis testing included 63 teams and 264 members.

#### Measures

##### EORs

We used the same scale in Study 1 to measure OI and EC, and their Cronbach’s alphas were 0.90 and 0.90, respectively. We also used the same method to create four EOR categories, including 28 quasi-spot contract, 8 underinvestment, 12 overinvestment, and 24 mutual investment approaches (see Table 7).

**TABLE 7:** Study 2: EOR categories.

	EOR: team level					ANOVA	
	Quasi-spot contract	Under investment	Over investment	Mutual investment	Total mean	*F*-value	*p*-value
Offered inducements	3.7526 (−0.8808)	4.0446 (−0.5931)	5.2202 (0.5648)	5.6042 (0.9430)	4.6468	52.91	0.000
Expected contributions	4.7527 (−0.8403)	6.0673 (0.7090)	5.3077 (−0.1863)	6.2276 (0.8979)	5.9346	37.48	0.000
N of team	28	8	12	24	72		

*Numbers in parentheses are means of standardized scores.*

##### Collective efficacy

We used the same scale to measure collective efficacy. However, in Study 2, we asked the team members to rate their perception of team collective efficacy. Cronbach’s alpha was 0.94. All the teams’ Rwg was equal to or greater than 0.79, and the average was 0.97. ICC(1) was 0.13, while ICC(2) was 0.37. Finally, we aggregated the member-rated collective efficacy into the team level.

##### Team performance

The team performance measurement was the same as that in Study 1. In Study 2, Cronbach’s alpha was 0.90.

##### Control variables

We controlled for team size, average age, male percentage, average education level, and the average tenure of team members.

#### Analyses

We used STATA to analyze the data in Study 2, including descriptive statistics, correlation analysis, and regression.

#### Results

Table 8 shows the correlations and descriptive statistics of Study 2. All binary correlations between the mutual investment EOR, team collective efficacy, and team performance were positive.

**TABLE 8:** Study 2: Correlations and descriptive statistics.

	Mean	*SD*	(1)	(2)	(3)	(4)	(5)	(6)	(7)	(8)	(9)	(10)
(1) Team size	4.19	2.54										
(2) Average age	33.51	4.55	0.02									
(3) Male percentage	0.59	0.34	–0.02	0.25^∗^								
(4) Average education	4.27	0.48	−0.45^∗∗∗^	–0.05	–0.15							
(5) Average team tenure	6.15	4.47	0.28^∗^	0.62^∗∗∗^	0.01	−0.28^∗^						
(6) Quasi-spot contract	0.40	0.49	0.12	–0.16	–0.05	0.06	–0.10					
(7) Underinvestment	0.11	0.32	0.07	0.04	0.06	–0.10	–0.03	−0.29^∗^				
(8) Overinvestment	0.17	0.38	–0.08	0.12	0.15	0.16	–0.08	−0.37^∗∗^	–0.16			
(9) Mutual investment	0.32	0.47	–0.11	0.04	–0.12	–0.12	0.19	−0.55^∗∗∗^	−0.24†	−0.31^∗^		
(10) Team collective efficacy (Time 2)	4.98	0.54	0.10	0.14	–0.02	–0.00	–0.04	−0.28^∗^	–0.01	0.14	0.19	
(11) Team performance (Time 3)	3.74	0.74	0.01	–0.17	–0.14	–0.14	–0.00	−0.45^∗∗∗^	0.14	–0.06	0.43^∗∗∗^	0.28^∗^

*p* < 0.1, *p < 0.05, **p < 0.01, and ***p < 0.001; *N* = 63.

Table 9 shows the results of the hierarchical regression analysis. Step 2 shows that the mutual investment approach was positively related to team collective efficacy (β = 0.40, *p* < 0.05) compared with the quasi-spot contract approach, whereas the underinvestment and overinvestment approaches were not significantly related to team collective efficacy. Thus, Hypothesis 1 is supported.

**TABLE 9:** Study 2: Hierarchical regression analysis results.

	Team collective		Team performance		
	efficacy (Time 2)		(Time 3)		
	Step 1	Step 2	Step 3	Step 4	Step 5
Constant	4.99^∗∗∗^	4.80^∗∗∗^	3.73^∗∗∗^	3.28^∗∗∗^	3.34^∗∗∗^
Team size	0.04	0.05^†^	–0.03	0.01	–0.01
Average age	0.04^†^	0.04^†^	–0.04	–0.04	−0.05^†^
Male percentage	–0.16	–0.14	–0.25	0.17	–0.13
Average education	–0.01	0.03	–0.29	–0.13	–0.14
Average team tenure	−0.04^†^	−0.04^†^	0.02	0.01	0.02
**EOR (Time 1)**					
Underinvestment		0.14		0.75^∗^	0.71^∗^
Overinvestment		0.31		0.44^†^	0.35
Mutual investment		0.40^∗^		0.89^∗∗∗^	0.77^∗∗∗^
Team collective efficacy					0.29^†^
(Time 2)					
R^2^	0.08	0.18	0.08	0.34^∗∗^	0.38^∗∗^
ΔR^2^		0.10^†^		0.26^∗∗∗^	0.04^†^
F	0.98	1.49	0.98	3.49^∗∗^	3.58^∗∗^
ΔF		2.23^†^		7.15^∗∗∗^	3.14^†^

^†^*p* < 0.1, *p < 0.05, **p < 0.01, and ***p < 0.001; *N* = 63. $\Delta \,F=\left(\Delta \,R^{2}/\Delta \,k\right)\,\left(N-k_{2}-1\right)/\left(1-R_{2}^{2}\right)$, where *k* is the number of predictors, and *N* is the sample size (Jaccard et al., 1990).

Step 4 in Table 9 illustrates that the underinvestment, overinvestment, and mutual investment EOR approaches are positively related to team performance (β = 0.75, *p* < 0.05; β = 0.44, *p* < 0.10; and β = 0.89, *p* < 0.001, respectively). When entering the equation (Step 5), collective efficacy was positively and marginally significantly related to team performance (β = 0.29, *p* = 0.08), which may result from the small sample size. Meanwhile, all coefficients of underinvestment, overinvestment, and mutual investment approaches on team performance decreased, and the coefficient of the overinvestment approach was non-significant. Table 10 also shows the indirect effect of mutual investment EOR on team performance through collective efficacy. These results indicate that team collective efficacy mediates the relationship between EOR approaches and team performance. Thus, Hypothesis 2 is partially supported.

**TABLE 10:** Study 2: Mediation test using Monte Carlo Method–Hypothesis 2.

				90% confidence	
	a	b	a × b	interval	
Underinvestment	0.14	0.29	0.04	−0.07	0.18
Overinvestment	0.31	0.29	0.09	−0.01	0.25
Mutual investment	0.40	0.29	0.12	0.00	0.28

*The mediator is team collective efficacy.*

#### Discussion

Through a rigorous research design with multi-source and multi-time data collection, Study 2 shows similar relationship patterns between EOR approaches, collective efficacy, and team performance. The results partially replicate the findings of Study 1 that EOR approaches will influence team performance by shaping team collective efficacy when investigating the effect of EORs from a social cognitive perspective.

## Discussion

In this study, we extend the EOR literature by diverging from the focus on relationships among social agents and departing from the focus on overall EOR practices. Instead, we focus on the characteristics of the agents and on specific team-level HRM practices. Drawing on social cognitive theory, we find that collective efficacy mediates the relationship between team EOR approaches and performance. Furthermore, team cohesion is a boundary condition for team collective efficacy effects on team performance, thereby revealing that collective efficacy has limitations.

### Theoretical Contributions

First, we contribute to the EOR literature by exploring a new mechanism between EORs and team performance. We go beyond the social relationship perspective and shift attention to the social cognition of work teams to link team-level EOR approaches to team performance. Our study suggests that OI and EC jointly shape team collective efficacy. Therefore, the mutual investment EOR approach, which offers high inducements and expects high contributions, is optimal for generating desirable team performance through team collective efficacy. This new insight enriches the EOR literature by understanding the effects of EOR approaches from the perspective of relationships among agents and the characteristics of agents. Moreover, our study is also a response to the call to examine the role of cognitive processes or states between EORs as environment stimuli and team behavioral responses and outcomes (Jia et al., 2014).

Second, we hypothesize a team-level moderated mediation model to link EORs to team performance. In doing so, we enrich EOR studies that have largely treated EORs as firm-level HRM practices that are linked to macro-level firm performance (Wang et al., 2003) or micro-level individual performance and attitudes (Shaw et al., 2009). Different employment relationship approaches coexisting in a single firm has become a norm. In addition, our research suggests that an organization can use multiple EOR approaches across teams to effectively balance its paradoxical need for employment flexibility and employee commitment, which is a fundamental problem in employment practice and research (Tsui et al., 1995; Tsui and Wang, 2002).

Third, we also contribute to social cognitive theory through the contingent understanding of collective efficacy. Collective efficacy influences performance only in cultures that ascribe to collectivistic values (Schaubroeck et al., 2000; Khong et al., 2017). Meanwhile, meta-analytic evidence has shown that collective efficacy has inconsistent effect sizes on team performance (Gully et al., 2002; Stajkovic et al., 2009), thereby motivating us to explore contingent factors. We introduce team cohesion, which is a culture-free construct with collective meanings, as a moderator and find that collective efficacy positively relates to team performance under high team cohesion, whereas this relationship is not significant under low team cohesion. The results indicate that collective efficacy will not consistently lead to high performance.

### Practical Implications

Our study has implications for practitioners. First, team supervisors can improve team performance by adopting mutual investment EOR approaches, which can help team members build collective efficacy. Second, team supervisors should use their discretion to offer inducements and expect contributions according to team functions and significance to attain optimal performance, despite top-level managers having overall authority in making HRM-policy decisions. Third, EOR approaches that build high team collective efficacy do not consistently ensure high team performance. Instead, team supervisors should encourage high-level cohesion to trigger the positive effect of team collective efficacy.

### Limitations and Future Research Directions

First, we adopt the social cognitive perspective to explore how team-level EORs influence team performance by shaping the states and characteristics of teams and introduce collective efficacy as a representative variable. However, team effectiveness also requires skills (Bandura, 1997). Future researchers can explore the interaction between efficacy beliefs and skills in shaping workplace outcomes. In addition, future researchers should consider other social agent states and characteristics, such as psychological safety.

Second, we organize our logic based on Bandura (1997, 2001, 2009), that collective efficacy and self-efficacy have similar sources. Nevertheless, collective efficacy and self-efficacy are found to be homologous in the lab but not in the field (Chen et al., 2002). Thus, we recommend future studies to simultaneously test team EORs for effects on team members’ self-efficacy and team collective efficacy.

Third, a limitation of Study 1 involves our use of only supervisor ratings for all core variables, which may cause common method bias. We collected data at different times to minimize the problem. Although researchers have pointed out that observing significant interaction effects with common source data is difficult, which means the conservation of our findings (Siemsen et al., 2010; Rego et al., 2017), we still conducted Harman’s single-factor test and a full collinearity test. The results of both methods show that our model does not suffer from CMV. In addition, we asked team members to rate collective efficacy at Time 2 in Study 2 to decrease CMV. Thus, we recommend that future researchers address this problem with a more rigorous design.

Fourth, we could not replicate the full theoretical model in Study 2. We did not include team cohesion, as the aim of Study 2 was to address the common method issues of Study 1, and the CMV problem need not be considered when testing interaction effects (Siemsen et al., 2010). Meanwhile, the participants of Study 2 answered the questionnaires on their smartphones. We had to keep the questionnaires as short as possible owing to participants’ limited attention span while on smartphones. Therefore, we tried to replicate only the most important part of the theoretical model, that is, the indirect effect of team-level EORs on team performance through collective efficacy. Thus, Study 1 and Study 2 have obvious advantages and disadvantages. However, they complementarily support our theoretical model. Hence, future researchers should collect additional comprehensive data sets to address these limitations.

## Conclusion

EORs have been shown to affect performance, employee attitudes, behaviors, and team creativity from social exchange (Tsui et al., 1997; Shaw et al., 2009), social embeddedness (Hom et al., 2009), and social structural perspectives (Jia et al., 2014). We shift from these relational perspectives to the cognitive perspective. Focusing on the team level, we show that the mutual investment EOR approach, which offers high inducements and expects high contributions, is superior in generating team collective efficacy and performance. In addition, we show that team cohesion strengthens the effect of collective efficacy on team performance, thereby strengthening the effect of the mutual investment EOR approach on team performance. Consequently, we suggest that firms adopt mutual investment HRM practices and improve team cohesion.

## Data Availability Statement

The datasets generated for this study are available on request to the corresponding author.

## Ethics Statement

The studies involving human participants were reviewed and approved by the Science & Technology Research Office of Nanjing University. The patients/participants provided their written informed consent to participate in this study.

## Author Contributions

JL and LJ are responsible for idea generation. JL and YC wrote the manuscript. LJ implemented the study. HK revised the manuscript. JL and SY carried out the data analysis.

## Conflict of Interest

The authors declare that the research was conducted in the absence of any commercial or financial relationships that could be construed as a potential conflict of interest.

## Supplementary Material

The Supplementary Material for this article can be found online at: https://www.frontiersin.org/articles/10.3389/fpsyg.2020.00206/full#supplementary-material
