# Supplementary material for: Employee–Organization Relationships and Team Performance: Role of Team Collective Efficacy
Source: Front Psychol. 2020 Mar 6;11:206. doi: 10.3389/fpsyg.2020.00206 (PMC7067981; doi:10.3389/fpsyg.2020.00206)
Supplement: Supplementary file 1 [file Table_1.DOCX]

**Appendix 1: Tests for response bias of Study 1**

|  | **M1** | **M2** | **Difference** | **t** | **95% Confidence Interval** | |
| --- | --- | --- | --- | --- | --- | --- |
| Team size | 7.403 | 7.815 | -.412 | -1.089 | -1.155 | .331 |
| Supervisor age | 4.033 | 3.970 | .063 | .446 | -.213 | .339 |
| Supervisor gender | .212 | .227 | -.016 | -.438 | -.086 | .055 |
| Supervisor education | 3.365 | 3.373 | -.009 | -.105 | -.169 | .152 |
| Supervisor company tenure | 9.069 | 9.365 | -.296 | -.456 | -1.571 | .979 |

Note: M1 = mean of the variable in the first wave; M2 = mean of the variable in the second wave; Difference = M1 – M2

**Appendix 2: Scales of core variables used in Study 1 and Study 2**

**Offered inducement**

To what extent does your firm provide the following inducements to the group of employees...

1. Value employees’ feedback on company’s overall policies.
2. Emphasize employee’s career development.
3. Care about employees’ satisfaction at work.
4. Create opportunities for employees to show their talents.
5. Treat each employee fairly.
6. Value employees’ suggestions on work.
7. Empower employees fully within their sphere of responsibility.
8. Encourage employees to participate actively in department- or firm-level decision-makings.
9. Respect human dignity.
10. Train employees on knowledge and skills for their jobs and career development.
11. Provide competitive salaries.
12. Provide generous housing subsidies besides the prescriptions by the law.
13. Provide competitive bonuses.
14. Offer good health care and medical insurance besides the prescriptions by the law.

(1 = seldom emphasized at all ~ 7 = emphasized very much, □ = not existing)

**Expected contribution**

To what extent does your firm emphasize the following expected contributions from the group of employees…

1. Fulfill its job inside and out.

2. Complete the performance goal in quality and quantity.

3. Do things legally and follow company’s policies and rules.

4. Conscientiously complete extra assignments at a moment’s notice.

5. Work seriously and accurately.

6. Team up with others in the job.

7. Work hard without complaints.

8. Contribute to the future development of the company or department.

9. Promote actively company’s image and reputation.

10. Take initiative to make constructive suggestions.

11. Adopt new ideas and methods actively to improve work.

12. Continuously improve work procedures and methods.

13. Take the initiative to carry out new or challenging assignments.

(1 = seldom emphasized at all ~ 7 = emphasized very much, □ = not existing)

**Collective efficacy**

Overall, the group of employees as a whole…

1. Is very effective.
2. Some members in this group can do their jobs well.
3. I feel confident about the collective capability of this group to perform the tasks very well.
4. Is able to solve difficult tasks if we invest necessary effort.
5. I feel confident that this group as a whole will be able to manage effectively unexpected troubles.
6. Is totally competent to perform the tasks.
7. Is able to allocate and integrate available resources to perform the tasks well.
8. Knows how to transform members’ abilities into group capabilities to do the tasks well.

(1 = strongly disagree ~ 6 = strongly agree)

**Team cohesion**

Overall, the group of employees as a whole…

1. Is ready to defend each other when facing criticism by outsiders.

2. Helps each other well on the job.

3. Gets along well with each other.

4. Sticks together well.

(1 = strongly disagree ~ 6 = strongly agree)

**Team performance**

Overall, the group of employees as a whole…

1. The quantity of work

2. The quality of work

3. The work planning and allocation

4. The knowledge of tasks

5. Overall performance

(1 = below average, 2 = somewhat below average, 3 = about average, 4 = somewhat above average, 5 = above average)
